# Backward Dislocation of the Carpus

**Published:** 1844-11-02

**Authors:** 


BACKWARD DISLOCATION OF THE CARPUS.

David B—, aged eighteen, admitted under Mr. Scott, Sept. 4th. He had fallen from the mainmast of a brig, and besides having driven in the lower part of the outer wall of the frontal bone, in the situation of the sinus, and dislocated the middle finger of the right hand, had also displaced the left carpus backwards, the dislocation being evidenced by the following symptoms:—Lower ends of the radius and ulna prominent, both styloid processes being entire, a marked depression beneath, or rather in front of them. Hand partially adducted and thrown backwards; a prominent tumour, formed by the carpus, extending from the level of the radius and ulna backwards, i. e., towards the elbow, for about an inch and a half; a marked depression above the tumour, i. e., towards the elbow; movements of the hands almost lost, there remaining only slight power of extension, none of flexion. No inconvenient symptom resulted subsequent to reduction, which was effected by making extension from the hand, the elbow being fixed by the knee.—*Ibid.*

